# Meeting new *Canadian 24-Hour Movement Guidelines for the Early Years* and associations with adiposity among toddlers living in Edmonton, Canada

**DOI:** 10.1186/s12889-017-4855-x

**Published:** 2017-11-20

**Authors:** Eun-Young Lee, Kylie D. Hesketh, Stephen Hunter, Nicholas Kuzik, Ryan E. Rhodes, Christina M. Rinaldi, John C. Spence, Valerie Carson

**Affiliations:** 1 Faculty of Physical Education and Recreation, University of Alberta, 8840-114 Street Van Vliet Complex, Edmonton, AB T6G 2H9 Canada; 2 Institute for Physical Activity and Nutrition, School of Exercise and Nutrition Sciences, Faculty of Health, Deakin University, Geelong, VIC 3220 Australia; 3 School of Exercise Science, Physical and Health Education, University of Victoria, Victoria, BC V8W 2Y2 Canada; 4 Department of Educational Psychology, Faculty of Education, University of Alberta, Edmonton, AB T6G 2G5 Canada

**Keywords:** Physical activity, Sedentary behaviour, Sleep, Guidelines, Toddlers, Body mass index

## Abstract

**Background:**

Canada has recently released guidelines that include toddler-specific recommendations for physical activity, screen-based sedentary behaviour, and sleep. This study examined the proportions of toddlers meeting the new *Canadian 24-Hour Movement Guidelines for the Early Years (0–4 years)* and associations with body mass index (BMI) z-scores in a sample from Edmonton, Canada.

**Methods:**

Participants included 151 toddlers (aged 19.0 ± 1.9 months) for whom there was complete objectively measured physical activity data from the Parents’ Role in Establishing healthy Physical activity and Sedentary behaviour habits (PREPS) project. Toddlers’ physical activity was measured using ActiGraph wGT3X-BT monitors. Toddlers’ screen time and sleep were measured using the PREPS questionnaire. Toddlers’ height and weight were objectively measured by public health nurses and BMI z-scores were calculated using World Health Organization growth standards. Meeting the overall *24-Hour Movement Guidelines* was defined as: ≥180 min/day of total physical activity, including ≥1 min/day of moderate- to vigorous-intensity physical activity; no screen time per day (for those aged 12–23 months) or ≤1 h/day of screen time per day (ages 24–35 months); and 11–14 h of sleep per 24-h period. Frequency analyses and linear regression models were conducted.

**Results:**

Only 11.9% of toddlers met the overall *24-Hour Movement Guidelines*, but this finding was largely driven by screen time. The majority of toddlers met the individual physical activity (99.3%) and sleep (82.1%) recommendations, while only 15.2% of toddlers met the screen time recommendation. No associations were observed between meeting specific and general combinations of recommendations within the guidelines and BMI z-scores.

**Conclusions:**

Most toddlers in this sample were meeting physical activity and sleep recommendations but were engaging in more screen time than recommended. Consequently, only a small proportion of toddlers met the overall guidelines. Based on the findings of this study, identifying modifiable correlates of screen time to inform appropriate strategies to reduce screen time appears key for increasing the proportion of toddlers meeting the *24-Hour Movement Guidelines for the Early Years*. Future research should examine the associations between meeting the new guidelines and other health indicators. Furthermore, future high-quality studies examining dose-response relationships between movement behaviours and health indicators are needed to inform guideline updates.

## Background

One assumption regarding children in the early years (aged 0–4 years) is that they are inherently physically active and thus healthy [1]. Though several studies have consistently reported that physical activity is favourably associated with several health outcomes [2–8], the amount that early years children participate in varies significantly across studies [9]. As for sedentary behaviour, television viewing,which is the most commonly assessed type of sedentary behaviour, is consistently associated with unfavourable health outcomes [2, 3, 5–8, 10, 11]. Given that physical activity and sedentary behaviour patterns can be established in the early years [12, 13], it is important to promote healthy active lifestyles during these formative years. As such, Canada developed Physical Activity and Sedentary Behaviour Guidelines for the Early Years in 2012 [14, 15]. These guidelines aligned with those developed in Australia and the United Kingdom [16, 17].

In addition to separate physical activity and sedentary behaviour guidelines, sleep guidelines have been developed by several organizations, including the National Sleep Foundation [18] and the American Academy of Sleep Medicine [19]. These guidelines are based on evidence that adequate sleep is important for the health of children in the early years [18, 19]. There has been recent scientific recognition that physical activity, sedentary behaviour, and sleep are co-dependent behaviours that form a movement behaviour continuum within a 24-h period, with mutual influence on health [20]. Consequently, Canada has recently taken a unified approach and developed *Canadian 24-Hour Movement Guidelines for the Early Years (0–4 years): An Integration of Physical Activity, Sedentary Behaviour, and Sleep* [21]. These guidelines were informed by four systematic reviews on the association between physical activity [5], sedentary behaviour [6], sleep [7], and combinations of movement behaviours [8] with health indicators in infants, toddlers, and preschoolers, in conjunction with novel compositional analyses, expert opinion, international harmonization, and stakeholder input.

Determining the proportions of children who meet or do not meet the new *24-Hour Movement Guidelines*, and specific recommendations within those guidelines, enables researchers and public health practitioners to more effectively monitor participation and identify health risks. This knowledge is important from a population health standpoint because it can inform the research and development of appropriate preventive strategies, such as interventions. This knowledge can also aid the informed allocation of limited public resources and the design of public and professional services [22, 23]. Furthermore, it is important to examine associations between meeting/not meeting guidelines and health indicators, to validate existing recommendations for support of guideline adoption.

Prior to the integrated approach taken by the new *24-Hour Movement Guidelines*, adherence to individual physical activity and sedentary behaviour guidelines among the toddler age group (aged 12–35 months) has been reported in only a limited number of studies [24–27]. Since available evidence is limited in quantity, and measurement of the behaviours has varied (e.g., accelerometry brands, data reduction decisions, accelerometer cut-points, subjective vs. objective measures), compliance to physical activity and sedentary behaviour guidelines differs significantly across studies and population groups. For instance, the prevalence of meeting physical activity recommendations has ranged from 0.4 to 97.5% across four studies [24–27]. Additionally, adherence to the screen time recommendation within the sedentary behaviour guidelines has ranged from 18.8 to 50.0% between two studies [25, 27]. For sleep, toddlers meeting the sleep recommendations from either the National Sleep Foundation [18] or the American Academy of Sleep Medicine [19] ranged from 66.2 to 85.9% among two studies [28, 29].

Apart from one other paper in this supplement issue [30], no evidence exists on the associations between meeting movement guidelines (i.e., physical activity, sedentary behaviour, sleep) and adiposity in toddler-only samples. The evidence gaps as well as the disintegrated focus on physical activity, sedentary behaviour, and sleep make it difficult for researchers to have the holistic understanding of children’s behavioural patterns and contexts within a 24-h period that are relevant for optimal health and development. Therefore, the primary objectives of this study were to: (1) determine the proportions of toddlers achieving different combinations of the recommendations within the new *24-Hour Movement Guidelines,* and (2) examine the associations between meeting different combinations of the recommendations within the guidelines and adiposity in a sample from Edmonton, Canada. A secondary objective was to determine prevalence estimates of physical activity, sedentary behaviour, and sleep.

## Methods

### Participants

This study used baseline data from the Parents’ Role in Establishing healthy Physical activity and Sedentary behaviour habits (PREPS) project. In partnership with the local health authority (Alberta Health Services), parents and their toddlers (aged 12–23 months) were recruited during 18-month immunization appointments at four large public health centres located in socio-economically diverse neighborhoods in Edmonton, Alberta, Canada. To be eligible for the PREPS study, toddlers had to be walking, and parents had to be able to speak and read English. Out of 491 eligible families, a total of 257 agreed to participate in the current study (participation rate: 52.0%). The reasons for declining to participate in the study included busy schedules/lack of time/fatigue (*n* = 74), no interest (*n* = 64), parental perception that their child would not wear the accelerometer belt (*n* = 60), travel/illness/moving away (*n* = 20), or a parent not being present at the time of data collection (*n* = 16).

### Procedures

Data were collected from October 2014 to December 2015. Consent forms and questionnaires were completed by eligible families who agreed to participate during the 15-min waiting period required after children’s immunizations. To minimize missing data, research staff checked the completeness of questionnaires at the appointment and asked parents to fill out any missing information, or contacted families via email or phone to follow up after the appointment. In both instances, parents were not required to answer any question they did not want to. At the immunization appointment, participating parents were also provided an accelerometer with verbal and written instructions for their toddler to wear it for seven consecutive days, except for overnight sleep and water-based activities (e.g., swimming, bathing). A pre-paid courier envelope was provided to return the accelerometer. Participating parents received a mid-week reminder about the continuous wear of their child’s accelerometer. Informed written consent was completed by a parent of each child who agreed to participate in the study. Ethics approval was granted by the University of Alberta Human Research Ethics Board.

### Measures

#### Objectively measured physical activity and sedentary time

Physical activity and sedentary time were measured objectively using waist-worn ActiGraph wGT3X-BT (ActiGraph Corp, Pensacola, FL, USA) accelerometers. Data were collected in 15-s epochs and non-wear time was defined as ≥80 consecutive 15-s intervals of zero counts (equivalent to ≥20 min of consecutive zero counts). It was assumed that daytime naps were included in non-wear time. Inclusion criteria for complete accelerometer data were at least 4 days with ≥1440 total 15-s intervals, which equates to ≥6 h of wear time [24, 31]. Per 15-s epoch, counts ranging from 0 to 24 were defined as sedentary time, 25–420 counts were defined as light-intensity physical activity (LPA), and >420 counts were defined as moderate- to vigorous-intensity physical activity (MVPA) [32, 33]. Minutes per day of sedentary time, LPA, and MVPA were calculated by dividing the number of 15-s intervals by four and then dividing by the total number of valid days. Wear-time standardized values were calculated by using the residuals obtained when regressing the variables on wear time [34], and these values were used in all data analyses.

Times spent in LPA and MVPA were added together to calculate total physical activity (TPA). Toddlers were classified as meeting the physical activity recommendation within the overall *24-Hour Movement Guidelines* if they accumulated an average of ≥180 min/day of TPA, including ≥1 min/day of MVPA [21]. The ≥1 min/day of MVPA definition was used to operationalize the recommendation that toddlers should accumulate “some” energetic play or MVPA within the 180 min/day of TPA. In addition to this MVPA recommendation for toddlers, preschoolers have an additional physical activity recommendation within the guidelines of participating in ≥60 min/day of MVPA. To determine if toddlers were progressing toward ≥60 min/day of MVPA, the proportion of toddlers accumulating an average of ≥20, ≥30, ≥45, and ≥60 min/day of MVPA was also calculated. No benchmark exists in the *24-Hour Movement Guidelines* for total sedentary time.

#### Screen time

In the PREPS questionnaire, parents reported how many hours and minutes their toddlers typically: (1) watched television, videos, or DVDs on a television, computer, or portable device; and (2) played video/computer games on electronic devices (e.g., a learning laptop, LeapFrog Leapster, computer, laptop, tablet, cellphone, the internet, PlayStation, or Xbox) per day during weekdays and weekend days. To calculate total screen time, weighted averages for weekday and weekend responses were computed ([weekday*5 + weekend*2]/7) for each television viewing and video/computer game use variable; weighted minutes per day of each variable were then summed. These questions were adopted from previous studies in the early years [35, 36] that have modified items from the Canadian Health Measures Survey [37] and have shown good 1-week test-retest reliability (intraclass correlation [ICC] = 0.82) in a sub-sample of toddlers who participated in the PREPS project [38]. Toddlers were classified as meeting the screen time recommendation within the overall guidelines if it was reported that they engaged in no screen time (for those aged 12–23-months) or ≤1 h/day of screen time (ages 24–35-months) [21].

#### Time spent restrained

In the PREPS questionnaire, parents reported the number of days per typical week that they limit the time their child spends being restrained (e.g., stroller, high chair, or car seat) to <1 h at a time. Response options ranged from 0 (never) to 7 (daily). This question was developed specifically for the PREPS study and had fair 1-week test-retest reliability (Kappa = 0.35) in a sub-sample of toddlers who participated in the PREPS project [38]. Toddlers were classified as meeting the ‘time spent restrained’ recommendation within the overall guidelines if it was reported that the time they spent being restrained was limited to <1 h at a time, 7 days a week.

#### Sleep duration

In the PREPS questionnaire, parents reported the time their toddlers usually spent sleeping during the daytime (i.e., nap) and nighttime. The total sleep time in hours was calculated by adding time spent in day- and nighttime sleep. These questions have shown good 1-week test-retest reliability (ICC = 0.78) in a sub-sample of toddlers who participated in the PREPS project (unpublished). Toddlers were classified as meeting the sleep recommendation with the overall guidelines if it was reported that they obtained 11–14 h of total sleep per 24-h period [21].

#### Canadian 24-hour movement guidelines for the early years (0–4 years)

As recommended for surveillance studies, toddlers were classified as meeting the overall *24-Hour Movement Guidelines* if they met the recommendations for physical activity (≥180 min/day of TPA, including ≥1 min/day of MVPA), screen time (no screen time for 12- to 23-month-olds and ≤1 h/day for 24- to 35-month-olds), and sleep duration (11–14 h/24-h period) [21]. Also in line with surveillance recommendations, sedentary time and time spent restrained were not considered components of meeting the overall *24-Hour Movement Guidelines* but were measured and reported for descriptive purposes [21]***.***

#### Body mass index (BMI) z-scores

Toddlers’ height and weight were objectively measured by public health nurses at the public health centre and reported by parents. BMI z-scores were calculated according to the World Health Organization (WHO) growth standards [39]. A BMI z-score ≥ 0.99 is defined as normal weight, 1.00 to 1.90 as at risk of becoming overweight, and >2.00 as overweight [40].

#### Covariates

Based on a previous study from the PREPS project examining the sociodemographic correlates of physical activity and sedentary behaviour [41], toddlers’ age, sex, race/ethnicity, main type of child care, and household income were included as covariates. Age (months) was computed from birthdates reported by parents in the PREPS questionnaire and data collection dates recorded by research staff. In the PREPS questionnaire, parents also reported toddlers’ sex (male or female); race/ethnicity (i.e., Aboriginal/First Nation, African-Canadian, Arabic, Asian/Pacific Islander, European-Canadian/Caucasian, Hispanic/Latino/Latina, or Other); hours per week spent in care other than parents (i.e., daycare centre, home daycare, another adult in your home, another adult outside your home, other); and gross household income over the past 12 months (<$25,000, $25,000–$50,000, $50,001–$75,000, $75,001–$100,000, >$100,000, and ‘do not know’). In line with a previous study in the current sample [41], toddlers’ race/ethnicity was categorized as European-Canadian/Caucasian and Non-European-Canadian/Non-Caucasian; household income was categorized as ≤$50,000, $50,001–$100,000, and >$100,000; and the main type of child care for each toddler was categorized as parental care (<4 h/week of non-parental), daycare centre (≥4 h/week in child-care centre and <4 h/week in any other care), home daycare (≥4 h/week in day home and <4 h/week in any other care), and other. For household income, two participants who did not respond to the question and five participants who responded “do not know” were excluded from analyses involving covariates.

### Statistical analysis

SAS version 9.4 (SAS Institute, Cary, NC, USA) was used to perform the statistical analyses. All continuous variables were checked for outliers (≥ ±3 standard deviations) [42]. As a result, values for three participants for sedentary time, one participant for MVPA, five participants for screen time, four participants for sleep, and seven participants for BMI z-score were truncated above or below ±3 standard deviations prior to analysis. A t-test for a continuous variable (i.e., toddlers’ age, screen time, total sleep time) and Chi-squared tests for categorical variables (i.e., toddlers’ sex and race/ethnicity, main type of child care, household income, time spent restrained) were performed to examine whether socio-demographic and toddlers’ behavioural characteristics differed between those included (*n* = 149) and excluded (*n* = 108) for the final analyses. Descriptive statistics, including means and standard deviations or percentages, were calculated for toddlers’ demographic characteristics, accelerometer-derived physical activity and sedentary time, and parental-reported screen time, sleep duration, and time spent restrained <1 h at a time. Frequency analyses were conducted to obtain the proportions of toddlers achieving specific (i.e., TPA, screen time, sleep, TPA + screen time, TPA + sleep, and screen time + sleep) and general combinations (i.e., all three, two out of three, one out of three, and none) of movement behaviour recommendations that were considered part of meeting the overall *24-Hour Movement Guidelines*. In addition, the proportions of toddlers achieving ≥20, ≥30, ≥45, and ≥60 min/day of MVPA were calculated. A series of linear regression models were then conducted to examine the associations between meeting specific and general combinations of movement behaviour recommendations within the guidelines and adiposity before and after adjusting for toddlers’ age, sex, race/ethnicity, main type of child care, and household income. Additional linear regression analyses were conducted to examine the association between sedentary time, time spent restrained for <1 h at a time (0–6 days vs. 7 days), and adiposity. BMI z-scores were normally distributed; thus, no transformation was made. Toddlers who did not meet recommendations served as the reference group. Statistical significance was set a priori at *p* < 0.05.

## Results

Out of 257 participants, 100 were excluded for either incomplete (i.e., <4 days of ≥6 h of wear time; *n* = 31) or no (*n* = 69) accelerometer data; four were excluded for a disability that might have an impact on physical activity; and two were excluded for being older than 35 months, leaving a total of 151 toddlers for the descriptive analyses. For the 151 toddlers, seven parents did not respond or responded “do not know” to the household income question and an additional 10 did not provide height and/or weight, leaving a total sample of 134 toddlers for the regression analyses, except for the analysis involving time spent restrained where the total sample was 133 toddlers. No significant difference was seen between samples of included and excluded participants in terms of age (Included: 19.0 ± 1.9 vs. Excluded: 19.8 ± 4.5), sex (47.0% vs. 51.4% females), and main type of child care (32.5% vs. 45.8% in parental care; 17.2% vs. 17.0% in child-care centre; 15.8% vs. 11.0% in day home; 34.5% vs. 26.3% in other). However, the sample of included participants had a larger percentage of toddlers with European-Canadian descent (59.6% vs. 42.5%) and families with higher household income (11.8% vs. 27.0% in ≤$50,000; 39.6% vs. 36.0% in $50,000–$100,000; 48.6% vs. 37.0% in >$100,000) compared to the sample of excluded participants. In terms of movement behaviours, significant differences between those included and excluded also existed for total screen time (85.2 ± 97.1 vs. 151.8 ± 209.7 min/day) and time restrained <1 h at a time for 7 days/week (34.0% vs. 16.4%), but not for total sleep (12.6 ± 1.3 vs. 12.3 ± 1.7 h/day). Additional participant characteristics for the included sample are presented in Table 1. The average time spent in LPA and MVPA was 240.2 ± 29.3 and 58.7 ± 18.7 min/day, respectively. In addition, toddlers spent an average of 316.7 ± 40.6 min/day in sedentary time. Further, among the sub-sample with BMI z-scores (*n* = 134), 29.1% were categorized as at risk for overweight and 11.9% were categorized as overweight (data not shown).

**Table 1:** Participant characteristics of toddlers living in Edmonton, Canada

	(n = 151)
Age (M ± SD)	19.0 ± 1.9 months
Sex (females, %)	47.0
Ethnicity/race (%)	
European-Canadian/Caucasian	59.6
Other^a^	40.4
Household income (*n* = 144) (%)	
> $100,000	48.6
$50,000 to $100,000	39.6
≤ $50,000	11.8
Main type of child care (%)	
Parental	32.5
Day care centre	17.2
Home day care	15.8
Other	34.5
Height (centimetres) (n = 140) (M ± SD)	82.2 ± 7.2
Weight (kilograms) (n = 140) (M ± SD)	11.2 ± 1.5
Body mass index z-score^b^ (*n* = 140) (M ± SD)	0.6 ± 1.2
Accelerometry data (M ± SD)	
Wear days	6.3 ± 1.0
Wear time (minutes/day)	10.3 ± 1.4
Physical activity (minutes/day) (M ± SD)	
Light-intensity physical activity	240.2 ± 29.3
Moderate- to vigorous-intensity physical activity	58.7 ± 18.7
Total physical activity	298.9 ± 40.9
Sedentary behaviour (minutes/day) (M ± SD)	
Sedentary time	316.7 ± 40.6
Television viewing	74.0 ± 82.5
Video game	11.2 ± 32.7
Total screen time (television viewing + video game)	85.2 ± 97.1
Restrained <1 h at a time/week (%) (*n* = 150)	
0 day	14.0
1 days	8.7
2 days	8.7
3 days	11.3
4 days	6.0
5 days	6.0
6 days	11.3
7 days	34.0
Sleep (hours/day) (M ± SD)	
Nap	2.0 ± 0.7
Night sleep	10.6 ± 1.4
Total sleep	12.6 ± 1.3

Values represent mean ± standard deviation (M ± SD) for continuous variables and percentage (%) for categorical variables

^a^Other included Aboriginal/First Nation, African-Canadian, Arabic, Asian/Pacific Islander, Hispanic/Latino/Latina, and others (self-expressed)

^b^BMI z-scores were calculated according to the World Health Organization (WHO) growth standards [38]

**Fig. 1:**
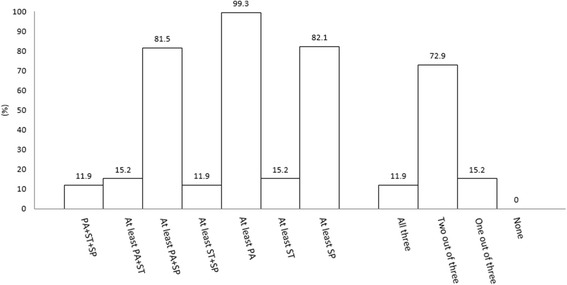
Proportion of toddlers meeting specific and general combinations of the *24-Hour Movement Guidelines* (*n* = 151). PA: physical activity; SP: sleep; ST: screen time. Meeting the recommendations is defined as ≥180 min/day of physical activity at any intensity, including ≥1 min/day of moderate- to vigorous-intensity physical activity; no screen time for <2-year-olds or ≤1 h/day for ≥2-year-olds; and 11–14 h of sleep/24 h

The proportions of toddlers achieving the specific and general combinations of movement behaviour recommendations within the guidelines are illustrated in Fig. 1. Specifically, 99.3% met the physical activity recommendations, 15.2% met the screen time recommendations, and 82.1% met the sleep recommendations. Additionally, 11.9% met both screen time and sleep recommendations, 15.2% met both physical activity and screen time recommendations, and 81.5% met both physical activity and sleep recommendations. Finally, 11.9% met the overall guidelines (i.e., all three recommendations), 72.9% met two out of three recommendations, and 15.2% met only one out of three recommendations. It is important to note that 148 out of 151 toddlers were aged 12–23 months and therefore the ‘no screen time’ recommendation applied.

**Fig. 2:**
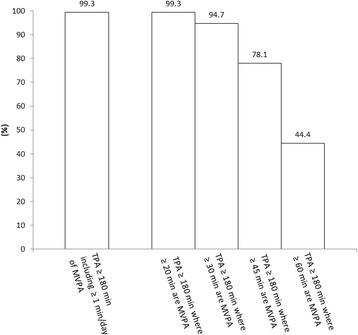
Proportion of toddlers progressing toward meeting the physical activity recommendations for preschoolers (n = 151). MVPA: moderate- to vigorous-intensity physical activity; TPA: total physical activity. Meeting the physical activity recommendations for toddlers is defined as ≥180 min/day of physical activity at any intensity, including ≥1 min/day of MVPA. Preschoolers have an additional physical activity recommendation within the guidelines of participating in ≥60 min/day of MVPA

Figure 2 presents the proportions of toddlers progressing toward meeting the physical activity recommendations for preschoolers (i.e., ≥60 min of MVPA daily). The proportions of toddlers accumulating an average of ≥180 min/day of TPA including an average of ≥20, ≥30, ≥45, and ≥60 min/day of MVPA were 99.3%, 94.7%, 78.1%, and 44.4% respectively.

Unadjusted and adjusted associations between meeting the specific and general combinations of movement behaviour recommendations and BMI z-scores are shown in Table 2. No significant associations were observed. Sedentary time and time spent restrained were also not associated with BMI z-score either before (*β* = −0.002; 95% CI = −0.007, 0.003; *β* = −0.034; 95% CI = −0.112, 0.045, respectively) or after (*β* = −0.001; 95% CI = −0.006, 0.005; *β* = −0.036; 95% CI = −0.120, 0.049, respectively) adjusting for covariates.

**Table 2:** Associations between meeting the combinations of the *24-Hour Movement Guidelines* and adiposity among toddlers (*n* = 134)

Meeting recommendations	BMI z-score	
	Unadjusted B (95% CI)	Adjusted B (95% CI)
Specific combinations of movement behaviours		
Physical activity + screen time + sleep		
Not met	Reference	Reference
Met	0.268 (−0.375, 0.910)	0.198 (−0.468, 0.863)
At least physical activity + screen time		
Not met	Reference	Reference
Met	0.181 (−0.400, 0.762)	0.047 (−0.559, 0.653)
At least physical activity + sleep		
Not met	Reference	Reference
Met	0.130 (−0.399, 0.659)	0.230 (−0.316, 0.776)
At least screen time + sleep		
Not met	Reference	Reference
Met	0.268 (−0.375, 0.910)	0.198 (−0.468, 0.863)
At least physical activity		
Not met	Reference	Reference
Met	0.919 (−1.434, 3.272)	0.391 (−2.064, 2.846)
At least screen time		
Not met	Reference	Reference
Met	0.181 (−0.400, 0.762)	0.047 (−0.559, 0.653)
At least sleep		
Not met	Reference	Reference
Met	0.087 (−0.451, 0.625)	0.222 (−0.339, 0.783)
General combinations of movement behaviours		
All three	Reference	Reference
Two out of three	−0.254 (−0.905, 0.398)	−0.175 (−0.851, 0.502)
One out of three	−0.335 (−1.139, 0.468)	−0.302 (−1.126, 0.522)
None	NA	NA

B (95% CI): unstandardized beta coefficients (95% confidence intervals)

BMI z-scores were calculated according to the World Health Organization (WHO) growth standards

Adjusted analyses included toddlers’ age, sex, race/ethnicity, household income and main type of child care as covariates

Meeting the recommendations is defined as ≥180 min of physical activity at varying intensities; no screen time for <2-year-olds or ≤1 h for ≥2-year-olds; and 11–14 h of sleep/day including nap during daytime

## Discussion

This study was the first to examine the proportion of children in the toddler age group meeting specific and general combinations of the newly developed *Canadian 24-Hour Movement Guidelines for the Early Years (0–4 years): An Integration of Physical Activity, Sedentary Behaviour, and Sleep* [21], and associations between meeting the new guidelines and BMI z-scores. This study also reported on prevalence estimates of sedentary time, the number of days restrained for <1 h at a time, and the accumulation of ≥20, ≥30, ≥45, and ≥60 min/day of MVPA. Only 12% of toddlers met the overall guidelines; however, 73% met two out of three recommendations, and all toddlers met at least one recommendation. In addition, most toddlers met TPA (99%) and sleep (82%) recommendations; however, only 15% met screen time recommendations. Almost half of the sample (44%) accumulated 60 min/day of MVPA, but only 34% of the sample were restrained for <1 h at a time daily. No associations existed between meeting specific and general combinations of recommendations, and BMI z-scores.

Almost all participants (99%) met the physical activity recommendations in our sample of toddlers. Using similar methodology and ActiGraph accelerometers, Hnatiuk and colleagues [24] as well as Santos and colleagues [30] also found that the majority of toddlers (90.5%–96.5%) met the physical activity recommendations. Additional research, using comparable methodology, is required to draw more conclusive results on toddlers’ physical activity. This includes accurately identifying different intensities of physical activity among toddlers using accelerometry. To date, research examining the validity and feasibility of accelerometers in toddlers is lacking compared to that of preschoolers [43]. It is also important to note that approximately 80% of the TPA that toddlers in the present study participated in was of light intensity. This is in line with existing evidence suggesting that most physical activity among toddlers is performed at low intensity [24, 37, 44].

LPA is indeed an important aspect of overall physical activity, given the behavioural characteristics of young developing children being spontaneous and intermittent [45]. In other words, physical activity tempo among young children under natural conditions is typically a series of brief bursts of intense activities scattered throughout varying intervals of lower-intensity activities [46]. This is likely because young developing children can recover quickly from a single burst of intense activity; however, they do not have the cardiovascular or neuromuscular capacity to maintain their activity at high intensity for a longer period [46, 47]. Regardless, MVPA appears to become increasingly important for healthy growth and development as early years children get older [48]. Thus, it is necessary to support families and child-care centres to ensure that all toddlers are progressing toward at least 60 min/day of MVPA throughout their preschool years. Such efforts may include providing environmental infrastructure for physical activity in neighbourhoods, implementing appropriate policies in child-care centres, and making resources available and accessible to families [49].

Similar to physical activity, a large proportion of toddlers (82%) met the sleep recommendations within the *24-Hour Movement Guidelines*. Among 202 Australian toddlers, 79.7% met the recommendation [30]. Using the same benchmarks as the *24-Hour Movement Guidelines* [18, 19], 66.0% of a national sample of New Zealand toddlers [50], 66.2% of 523 Italian toddlers [28], and 85.9% of 2800 Dutch toddlers [29] met the recommendations. Overall, these findings from a small number of studies suggest a number of toddlers from different population groups are achieving sufficient sleep. Given the limited number of studies, more research is required to elucidate prevalence estimates of toddlers obtaining adequate sleep for health benefits in various population groups. In regard to this, Beebe [51] suggested that when studying optimal sleep needs, future research should consider behavioural diversity across cultures as well as within a culture. This is because sleep behaviours and their correlates can be determined predominantly by cultural factors (e.g., parent-child co-sleeping, bed sharing). Further, some research findings suggest that daytime napping and nighttime sleep have different effects on health and development in early years children [52, 53]. Thus, it may be important to explore the health effects of daytime and nighttime sleep separately among early years children.

Despite the large proportion of toddlers meeting the physical activity and sleep recommendations, only 15% of toddlers met the screen time recommendation in the present study. This is similar or lower compared to other samples of toddlers in Canada (i.e., 21.9% in Vanderloo and Tucker’s work [25]; 50.0% in Botey et al.’s work [27]), and similar to estimates (11.4%) reported by Santos and colleagues [30] in a sample of Australian toddlers in this supplement issue. Therefore, findings across studies suggest a number of toddlers are engaging in more screen time than recommended. Of note, in the current study, only 15% of toddlers met both screen time and physical activity recommendations, and only 12% met both screen time and sleep recommendations. Therefore, the majority of toddlers in this sample engaged in the recommended amounts of physical activity and sleep but also spent more than the recommended amount of time in front of a screen. This is a concern when considering the combined effect of movement behaviours on health [54–57]. Given the negative associations observed between high screen time and health indicators (e.g., [10, 11, 58]), it is possible that engaging in more screen time than recommended could lessen the health benefits of engaging in sufficient physical activity and sleep in this age group.

In addition to screen time, the *24-Hour Movement Guidelines* include a recommendation of not being restrained for >1 h at a time or sitting for extended periods of time. It is not currently advised that this recommendation be included in the assessment of whether a toddler meets the overall guidelines; however, it can be incorporated in future efforts as evidence and measures continue to grow [21]. Currently, there are no other studies to compare data with the present study for time spent restrained for <1 h at a time. For example, in a sample of 542 Australian toddlers, the median time per day spent restrained was 30 min in a stroller or pram, 30 min in a car seat or capsule, and 60 min in a high chair or other chair [59] but it is not possible to determine whether this was continuous or accumulated time spent restrained per day. Therefore, further research is needed to confirm our prevalence findings in other population groups. In terms of total sedentary time, estimates in the present sample of toddlers (317 min/day) were lower than estimates in a national sample of 3- to 4-year-olds (348 min/day), 5-year-olds (381 min/day) [37], and 6- to 19-year-olds (516 min/day) [60]. That being said, similar to physical activity estimates, comparisons between studies should be made with caution because of different accelerometer brands and data reduction procedures between this study and the studies in older age groups.

The high prevalence of toddlers exceeding the screen time recommendation is the primary reason why only 12% of the sample met the overall guidelines. A similar finding was observed in a sample of Australian toddlers in this supplement issue, where only 8.9% met the overall guidelines [30]. Therefore, to increase the proportion of toddlers meeting the overall guidelines, research investigating the correlates and patterns of screen time in this age group is needed to inform health promotion strategies and interventions to reduce screen time. It is recommended that screen time be replaced with additional energetic play for greater health benefits within the guidelines [21]. More research is needed to understand the health impact of replacing screen time with other movement behaviours in this age group [8]. In older age groups of children, replacing screen time with MVPA was associated with positive health outcomes [61, 62]. In addition to screen time, it may also be important to identify modifiable correlates of prolonged time spent restrained.

No associations between meeting the specific and general combinations of guidelines and BMI z-score were observed in the present study. This finding is in line with a similar study in a sample of Australian toddlers [30], as well as four systematic reviews included in this supplement issue [5–8]. Specifically, none of the associations between meeting the guidelines and BMI z-score were significant among 202 Australian toddlers [30]. In addition, among four systematic reviews, the associations between physical activity and adiposity were predominantly null regardless of study design (i.e., experiment, observation) [5]. Additionally, associations between sedentary behaviour and adiposity were unfavourable and null [6]; the associations between sleep and adiposity were predominantly favourable, but null associations were also observed [7]. Furthermore, null and favourable associations were also observed between optimal combinations of movement behaviours and adiposity [8]. These findings are in contrast to more conclusive results on the relationship between movement behaviours and adiposity in older age groups [48, 63, 64].

Similar to previous research in early years children, measurement error of both behavioural and adiposity measures may explain why null findings were observed between meeting the guidelines and BMI z-scores in the present sample. Specifically, toddlers’ height and weight were objectively measured once by public health nurses and subsequently reported by parents, whereas typically it is standard in research to measure height and weight multiple times to minimize measurement error [65]. Additionally, screen time and sleep were parental-reported and, thus, recall and social desirability bias may have been present. Alternatively, it might simply be that it is difficult to observe conclusive associations among movement behaviours and adiposity in toddlers because it takes time for behavioural characteristics to lead to morbidity [66]. Future research using a longitudinal study design is needed to account for the temporal nature of the behaviour-morbidity relationships.

Though meeting the guidelines may not be important for adiposity in toddlers, guideline adoption may have an impact on other aspects of growth and development. For instance, toddlerhood is a period of rapid development in motor, sensory, cognitive, and social skills that bridges infancy and early childhood [67]. Thus, developmental indicators other than BMI z-scores may be more relevant to this age group. This is, in part, supported by three systematic reviews included in this supplement issue [5, 6, 8]. Compared to the adiposity indicator, more consistent associations were observed between physical activity and motor, cognitive, and psychosocial development [5]. Similar patterns were observed between sedentary behaviour and cognitive development [6], and between ideal combinations of movement behaviours and motor development [8]. Therefore, future studies should examine the associations between meeting the new guidelines and motor, cognitive, and psychosocial development in this age group. It is also important to note that the new *24-Hour Movement Guidelines for the Early Years* were developed to support healthy growth and development based on the best available evidence; however, specific benchmarks (e.g., 180 min/day of TPA) within the guidelines were determined primarily by lower-quality evidence [5–8, 21]. Therefore, future research should also continue to investigate the best physical activity, sedentary behaviour, and sleep thresholds for health benefits among children of the early years.

The main strength of this study was the use of a large sample of toddlers with objectively measured physical activity and sedentary time data relative to other previous studies involving Canadian toddlers [25, 27]. In terms of study limitations, screen time, sleep, and time spent restrained were measured subjectively via parental report; thus, measurement error may have been present. Nonetheless, the screen time and sleep measures have shown good reliability in toddlers [38]. In line with surveillance recommendations, only sleep duration was included in the analyses; other important aspects of sleep, including sleep quality, were not included. Furthermore, as previously discussed, there may have been measurement error associated with the objective height and weight measures because only one measurement was taken, and the measures were reported by parents and not by the nurses who took the measurements. In addition, approximately 42% of the sample was excluded from the analyses primarily due to incomplete or no accelerometer data, and some differences in demographic and movement behaviours existed between included and excluded participants, which may have impacted our findings. Specifically, it is likely that the proportion of toddlers meeting the screen time recommendation is overestimated, while time spent restrained for 7 days/week is underestimated. Another limitation of this study is the modest participation rate. Though the sample was recruited from multiple health-care sites in diverse neighbourhoods, the participation rate along with the differences between included and excluded participants may have impacted the generalizability of the findings, in particular for the descriptive data. Lastly, for consistency across movement behaviours, separate analyses were not conducted for weekdays and weekend days because this information was not available for all movement behaviours [21].

## Conclusions

An investigation of the proportions of toddlers meeting the new *Canadian 24-Hour Movement Guidelines for the Early Years* offers insights into healthy growth and development that can serve as an integral part of population health [68]. The findings of this study suggest that the majority of toddlers are meeting physical activity and sleep recommendations but engage in more screen time than recommended. Therefore, only a small proportion of the sample (12%) met the overall guidelines. Consequently, to increase the proportion of toddlers meeting the overall guidelines, it may be important to identify correlates and patterns of screen time among toddlers so appropriate strategies to reduce the time spent in front of a screen can be developed. Nonetheless, it should be noted that findings are based on a sample of toddlers living in Edmonton, Canada. Future work in representative samples of Canadian toddlers is needed to confirm these findings. In addition, though the associations between meeting the specific and general combinations of the *24-Hour Movement Guidelines* and BMI z-scores were null, replication is required using more rigorous study designs (i.e., experimental, longitudinal) with objectively measured movement behaviours, where possible, to confirm the findings. Furthermore, the association between meeting the guidelines and other health indicators should be examined. Finally, since the specific benchmarks within the new *Canadian 24-Hour Movement Guidelines for the Early Years* are based on lower-quality evidence, future high-quality studies are needed to provide further insight into the appropriate dose of movement behaviours for optimal health in the early years.

## Abbreviations

BMI: Body mass index
ICC: Intraclass correlation
LPA: Light-intensity physical activity
MVPA: Moderate- to vigorous-intensity physical activity
PREPS: Parents’ Role in Establishing healthy Physical activity and Sedentary behaviour habits
TPA: Total physical activity
WHO: World Health Organization
